# Morphological and Chemical Evaluations of Leaf Surface on Particulate Matter2.5 (PM2.5) Removal in a Botanical Plant-Based Biofilter System

**DOI:** 10.3390/plants10122761

**Published:** 2021-12-14

**Authors:** Yong-Keun Choi, Hak-Jin Song, Jeong-Wook Jo, Seong-Won Bang, Byung-Hoon Park, Ho-Hyun Kim, Kwang-Jin Kim, Na-Ra Jeong, Jeong-Hee Kim, Hyung-Joo Kim

**Affiliations:** 1 Department of Biological Engineering, Konkuk University, Seoul 05029, Korea; dragonrt@konkuk.ac.kr (Y.-K.C.); hjeda11@naver.com (H.-J.S.); jjw9802@naver.com (J.-W.J.); 2 Garden4u Co., Ansan 15524, Korea; garden4u_comp@naver.com (S.-W.B.); byonghpark@naver.com (B.-H.P.); 3 Department of Integrated Environmental System, Pyeongtaek University, Pyeongtaek 17869, Korea; ho4sh@ptu.ac.kr; 4 Urban Agriculture Research Division, National Institute of Horticultural and Herbal Science, Jeonju 54875, Korea; kwangjin@korea.kr (K.-J.K.); jnr202@korea.kr (N.-R.J.); kimjhee@korea.kr (J.-H.K.)

**Keywords:** *Ardisia japonica*, air quality, drought, roughness, wax

## Abstract

Particulate matter has been increasing worldwide causing air pollution and serious health hazards. Owing to increased time spent indoors and lifestyle changes, assessing indoor air quality has become crucial. This study investigated the effect of watering and drought and illumination conditions (constant light, light/dark cycle, and constant dark) on particulate matter2.5 (PM2.5) removal and surface characterization of leaf in a botanical plant-based biofilter system. Using *Ardisia japonica* and *Hedera helix* as experimental plants in the plant-based biofilter system, PM2.5, volatile organic carbon, and CO_2_, as the evaluators of indoor air quality, were estimated using a sensor. Morphological and chemical changes of the leaf surface (i.e., roughness and wax) associated with PM2.5 removal were characterized via scanning electron microscopy, Fourier transform infrared spectroscopy, and atomic force microscopy. The highest PM2.5 removal efficiency, stomata closure, high leaf roughness, and wax layer were observed under drought with constant light condition. Consequently, PM2.5 removal was attributed to the combined effect of leaf roughness and wax by adsorption rather than stomatal uptake. These results suggest that operating conditions of indoor plant-based biofilter system such as watering (or drought) and illumination may be applied as a potential strategy for enhancing PM2.5 removal.

## 1. Introduction

Particulate matter (PM) generated owing to rapid urbanization is increasing worldwide recently, and its presence in the environment causes air pollution (both indoor and outdoor). The PM with varying diameters (0.001–100 μm) have been designated by WHO (World Health Organization) as carcinogens; it engenders serious health effects such as respiratory and cardiovascular diseases in humans [1,2]. Higher exposure levels (approximately 2–5 times) to air pollution are reported indoors than outdoors [3]. Additionally, evaluating indoor air quality is crucial owing to increased time spent indoors (approximately 80–90%) and modern lifestyle changes [3,4]. Therefore, reducing indoor air pollution is of utmost significance.

Several approaches have been adopted to date, including ventilation and air conditioning systems, air purifiers containing high-efficiency particulate air (HEPA) filters, and plants to eliminate indoor PM. Among them, mechanical methods, namely ventilation and air purifier, have disadvantages, such as the loss of heat, high energy consumption, additional maintenance cost (i.e., exchange of filter), and reduced efficiency owing to extended use [2,3]. However, indoor botanical plants can overcome these drawbacks. Moreover, the elimination of major types of air pollutants (e.g., PM, volatile organic carbons (VOCs), CO_2_) by indoor botanical plants has been demonstrated in the previous study [3]. Indoor plants have various benefits such as promoting mental activity, enhancing productivity, and providing a comforting natural environment [4]. Hence, a plant-based biofilter system, which can be placed indoors to eliminate indoor PM, is currently gaining increasing attention. The plant-based biofilter system (e.g., varieties and density of plants, arrangement of system (e.g., hanging on walls), and adjustment of air circulation rate (i.e., associated with PM removal rate)) can be catered to the user’s requirement. Nevertheless, certain limitations exist, such as adequate selection of plant species, limited space for potted plants, and lower removal efficiency (RE) of PM. Many studies are focusing on the relationship between PM removal and plant characteristics, such as plant species and morphology of leaf and stem [5,6,7,8]. Thus, this study focuses on the effect of watering and illumination conditions on improving PM2.5 removal.

This study evaluates the ability of the indoor plant-based biofilter system to capture PM2.5 under drought stress with or without illumination. Consequently, the ecological modifications on the leaf surface and its association with PM2.5 capture are elucidated. In particular, the functional groups, roughness, and wax layer on the leaf surface are comprehensively assessed via Fourier transform infrared spectroscopy (FTIR), scanning electron microscopy (SEM), and atomic force microscopy (AFM). This study describes the morphological and chemical changes of the plant leaf surface under different operating conditions (i.e., watering and illumination) during the removal of indoor PM2.5.

## 2. Materials and Methods

### 2.1. Experiment Design for the Biofilter System Containing Indoor Plants

**Figure 1:**
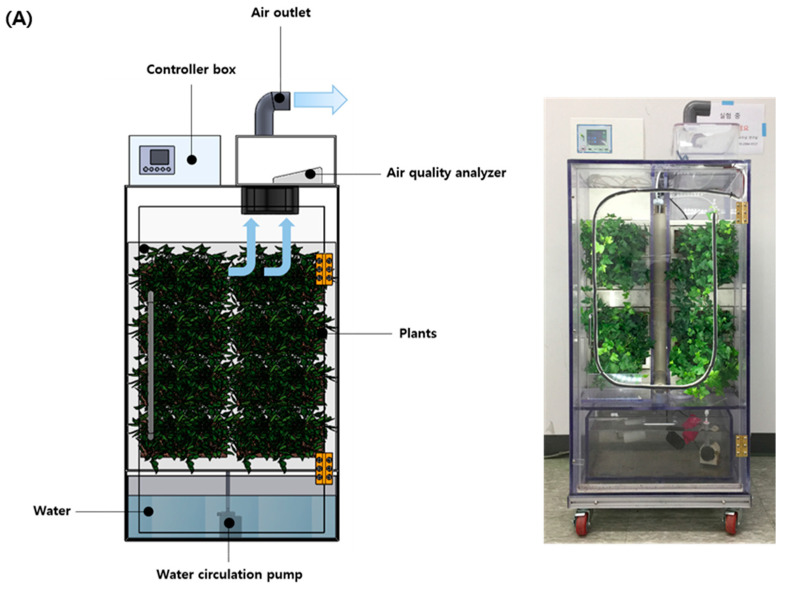

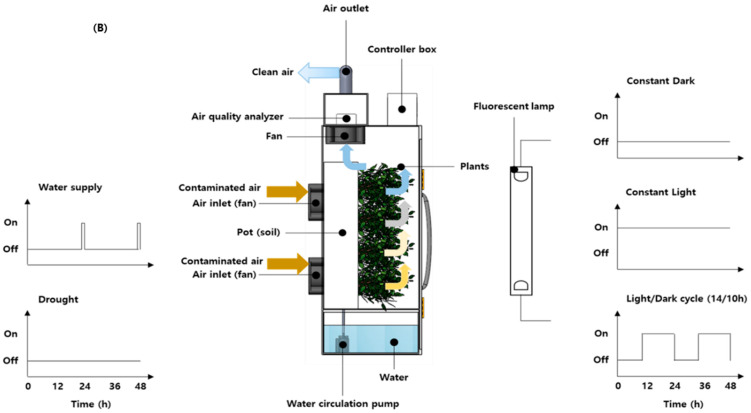
Schematic of (**A**) plant-based biofilter system and (**B**) different operating conditions (illumination and watering).

The schematic of plant-based biofilter system is shown in Figure 1. The dimension of the biofilter system was 40 cm (length) × 50 cm (width) × 70 cm (height), made by a transparent acrylic panel (total volume of 140 L). The set-up comprised individual pots (with dimensions of 10 cm (length) × 20 cm (width) × 12 cm (height), water container, water pump, fluorescent lamp for illumination, and fans (approximately 5 m/s) to introduce indoor air particles (i.e., real contaminated air including PM2.5, CO_2_, and VOCs) and release air from the biofilter (i.e., purified air). Soils in the individual pots contained a mixture (neutral pH) of organic soil (80%, *w*/*w*; Jeil, Seoul, Korea) and rice husk-derived commercial biochar (20%, *w*/*w*; Yougi Ind Co. Ltd., Jeolabuk-do, Korea). The used soils contained various nutrients such as N (720 ppm), P (40 ppm), K (1103 ppm), Ca (837 ppm), Mg (608 ppm), Fe (5082 ppm), Na (125 ppm), and Zn (12 ppm) [9]. Plant varieties namely *Ardisia japonica* and *Hedera helix*, used in this study, were purchased from a local flower market (Yangjae Flower Market, Seoul, Korea) and were adapted to indoor environment for seven days before the experiment. *A. japonica* was used for evaluating the removal of PM2.5, CO_2_, and VOCs under various watering (i.e., drought and water supply) and illumination conditions (i.e., the constant light (24 h), light/dark cycle (14 h:10 h), and constant dark (24 h)) in parallel or in series. In addition, *H. helix* was only used for comparing PM2.5 removal patterns with *A. japonica* under constant light with drought for 4 days and water supply for 4 days in parallel. The filter system without plants was also used for evaluating RE of PM2.5, CO_2_, and VOC, as control experiment. These plants were cultivated in the plant-based biofilter system under 24 μmol/m^2^/s light intensity (only under constant light and light/dark condition) at ambient temperature of 22–25 °C and relative humidity of 40–70% in a seminar room (Konkuk University, Republic of Korea). The experiments in plant-based biofilter system (i.e., with *A. japonica*) were performed under different watering and illumination conditions. Briefly, two types of watering conditions, of 1 L of water supply every day and drought (not supply water by watering stop), were considered; illumination conditions followed the constant light (24 h), light/dark cycle (14 h:10 h), and constant dark (24 h) represented as CL, LD cycle, and CD, respectively. The used water contained low cationic and anionic concentrations (e.g., Cl (<1 ppm) and Na (<1 ppm)). For the assessment of RE of PM2.5, CO_2_, and VOCs, the biofilter system containing *A. japonica* was initially subjected to water supply with CL, LC cycle, and CD illumination conditions for 10 days in parallel. Afterward, the biofilter system containing *A. japonica* was used for assessing RE of PM2.5, CO_2_, and VOCs, under drought with CL, LC cycle, and CD illumination conditions for 6 days in parallel. The experiment in serial was conducted for comparison with parallel. Briefly, the experiment was initially conducted under the drought condition for 4 days, and subsequently, water was supplied for 2 days at each illumination condition. *H. helix* was only used for comparing PM2.5 removal patterns under constant light with drought for 4 days and water supply for 4 days in parallel. All experiments were run in duplicate in serial. Briefly, the experiments were successively carried out after the first batch experiment to minimize variations.

### 2.2. Observation of A. japonica Leaf

After subjecting to drought and water supply conditions, the leaf of *A. japonica* was separated for analysis. The morphology of abaxial and adaxial leaf surface of lyophilized *A. japonica* separated after subjecting to different illumination (e.g., CL, LD cycle, and CD) and watering conditions was analyzed via SEM (TM4000 Plus, Hitachi Co., Tokyo, Japan). Additionally, the elemental compositions (e.g., C, O, S, Ca, and Pt) of adaxial surface layer and surface particle of *A. japonica* leaf were evaluated to demonstrate the compositions of captured particle owing to use of real PMs in the present study. The leaf of *A. japonica* was separated and then lyophilized at the end of experiment conducted under CL and drought condition. The harvested leaf was analyzed via SEM with energy-dispersive X-ray spectrometry (SEM/EDS; TM4000 Plus, Hitachi Co., Tokyo, Japan) in duplicate. The surface height maps and 3D profiles of adaxial surface of *A. japonica* leaf obtained under different illumination conditions (i.e., CL, LD cycle, and CD) with and without water were analyzed via AFM (XE-100, Park System, Suwon, Korea), and the functional groups on the adaxial surface were examined in the range of 600–4000 cm^−1^ via FTIR (FT/IR-4600 spectrometer, Jasco, Japan) to identify the wax layer on the leaf surface [8,10,11,12]. All experiments were run in duplicate.

### 2.3. Determination of Air Quality

The concentration of PM2.5, CO_2_, and VOCs were simultaneously analyzed using a commercial sensor-based instrument (Smart Aircok, Aircok Co., Seoul, Korea). The PM2.5 concentration and its RE were determined by the difference between the input and output air of the plant-based biofilter. The accumulated RE of PM2.5, CO_2_, and VOCs by the plant-based biofilter system can be expressed by Equations (1)–(3):

RE_acc_ of PM2.5 (%) = (PM2.5 _inp_ − PM2.5 _out_)/PM2.5 _inp_ × 100 (1)

RE_acc_ of CO_2_ (%) = (CO_2 inp_ − CO_2 out_)/CO_2 inp_ × 100 (2)

RE_acc_ of VOCs (%) = (VOCs _inp_ − VOCs _out_)/VOCs _inp_ × 100 (3)

### 2.4. Statistical Analysis

The Minitab 16.0 software (Minitab Inc., State College, PA, USA) was used for statistical analysis. We conducted one-way analysis of variance (ANOVA) to evaluate the significant differences in PM2.5 removal between the drought and water supply under each illumination condition (i.e., CL, LD cycle, and CD). The statistical significance was set as *p* < 0.05.

## 3. Results

### 3.1. Evaluation of PM2.5 Capture and Air Quality Using Biofilter System in Parallel

Figures S1 and S2 show the accumulated RE of PM2.5, CO_2_, and VOCs under CL, LD cycle, and CD conditions with water supply and drought in parallel. The accumulated RE of PM2.5 under CL and LD cycle decreased with water supply, whereas it increased with drought. The patterns of accumulated RE of VOCs were similar to those of PM2.5. In contrast, decrease in RE of CO_2_ was observed under overall illumination and watering conditions except under LD cycle with drought. In particular, RE of CO_2_ (7 to 0%) considerably decreased at initial days (0 to 2nd day) under CL with water supply and drought (Figures S1A and S2A); however, it greatly increased under LD cycle with water supply (0 day) and drought (2nd day) (Figures S1B and S2B). Although it was difficult to effectively assess the RE of CO_2_, the results implied that morphological and chemical changes in plant leaf influence the removal of PM2.5, CO_2_, and VOCs. The results show that watering and illumination conditions in the plant-based biofilter system likely influence PM2.5 removal, and thus, it can be considered one of potential strategies for enhancing PM2.5 removal. Hence, PM2.5 capture and air quality by the biofilter system was investigated in serial as elucidated in Section 2.2. In addition, the PM2.5 (approximately 8.8–10.4%) and VOCs (approximately 2.5–39.1%) were removed by the filter system without plants; however, no CO_2_ removal was observed (Figure S3). These results may indicate the effects on the filtration by soil and uptake by microorganisms in soil.

### 3.2. Influences of Drought on PM2.5 Capture and Air Quality Using Biofilter System in Serial

**Figure 2:**
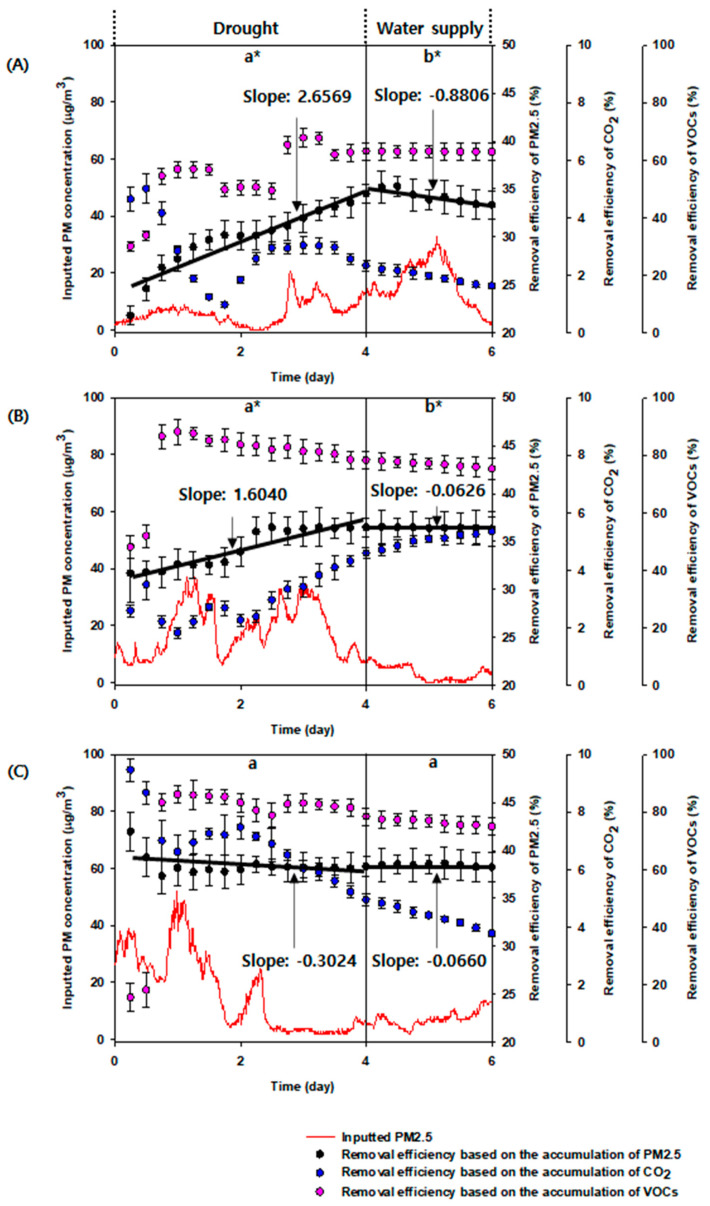
Removal efficiency of PM2.5, CO_2_, and VOCs by the plant-based biofilter system containing *Ardisia japonica* under (**A**) CL (continuous light), (**B**) LD cycle (light/dark cycle; 14 h/10 h), and (**C**) CD (continuous dark) with drought and with water supply in series. The error bars represent standard deviation. * is significance level of *p* < 0.05.

Based on the results shown in Figure 2A–C, the accumulated RE (slope: 2.6569) of PM2.5 was the highest under drought with CL, as compared to the other illumination conditions (LD cycle’s slope: 1.6040 and CD’s slope: −0.3024) under drought; however, the illumination conditions (i.e., CL, LD cycle, and CD) with water were negatively affected (CL’s slope: −0.8806, LD cycle’s slope: −0.0626, and CD’s slope: −0.0660). These results imply that the watering and illumination conditions in the plant-based biofilter system may influence the PM2.5 removal. The trend of accumulated RE of VOCs was similar to that of PM2.5 (Figure 2A,C). Although the stomata were closed during drought conditions, the increased VOC removal may be attributed to the presence or increase in wax on the leaf surface [13,14]. Briefly, the quantity and composition of wax can influence VOC removal during the closed stomata as reported in the previous studies [15,16]. In contrast, the pattern of CO_2_ RE varied with that of PM2.5 and VOCs. As shown in Figure 2A,C, the decrease in RE of CO_2_ associated with photosynthesis and respiration was observed under drought and only under CL and CD conditions. In addition, RE of CO_2_ decreased under water supply in serial (Figure 2A,C). However, a large increase and continuous increase in RE of CO_2_ were observed under LD cycle with drought and LD cycle with water supply, respectively, in serial (Figure 2B). The results imply that LD cycle, indicating photosynthesis and respiration, could be associated with RE of CO_2_ due to adaptation for plant growth and least influenced by the opening and closing of stomata and watering [17]. One-way ANOVA revealed that differences between drought and water supply for PM2.5 RE under CL and LD cycle conditions were significant (*p* < 0.002 and *p* < 0.012). However, the PM2.5 RE between drought and water supply under CD condition was not significant (*p* < 0.866) (Figure 2).

### 3.3. Morphological Evaluation of Leaf Surface on PM2.5 Removal

**Figure 3:**
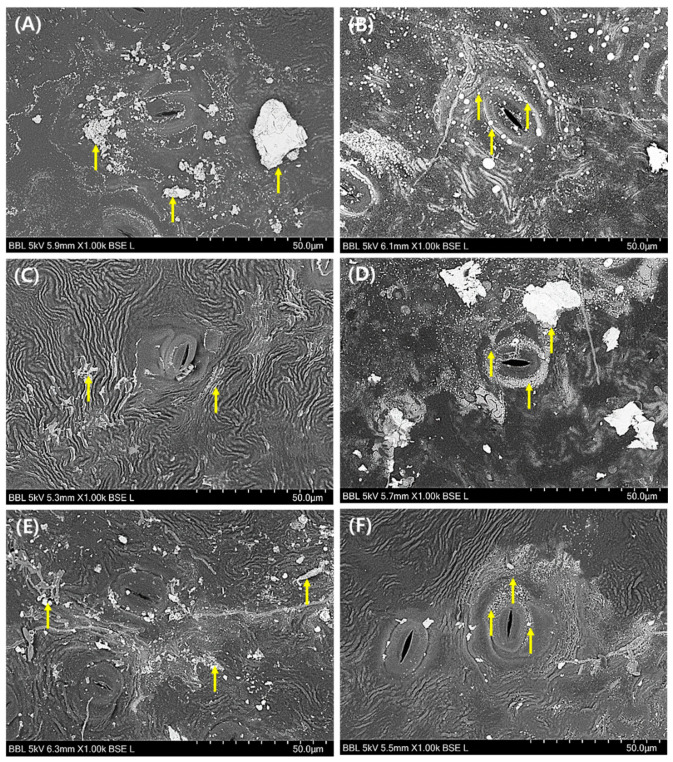
Scanning electron microscopy (SEM) images of abaxial surface of *Ardisia japonica* leaf under CL (continuous light) with (**A**) drought and (**B**) water supply, under LD cycle (light/dark cycle; 14 h/10 h) with (**C**) drought and (**D**) water supply, and under CD (continuous dark) with (**E**) drought and (**F**) water supply. Symbol (↑) indicates the distributed particulate matter.

**Figure 4:**
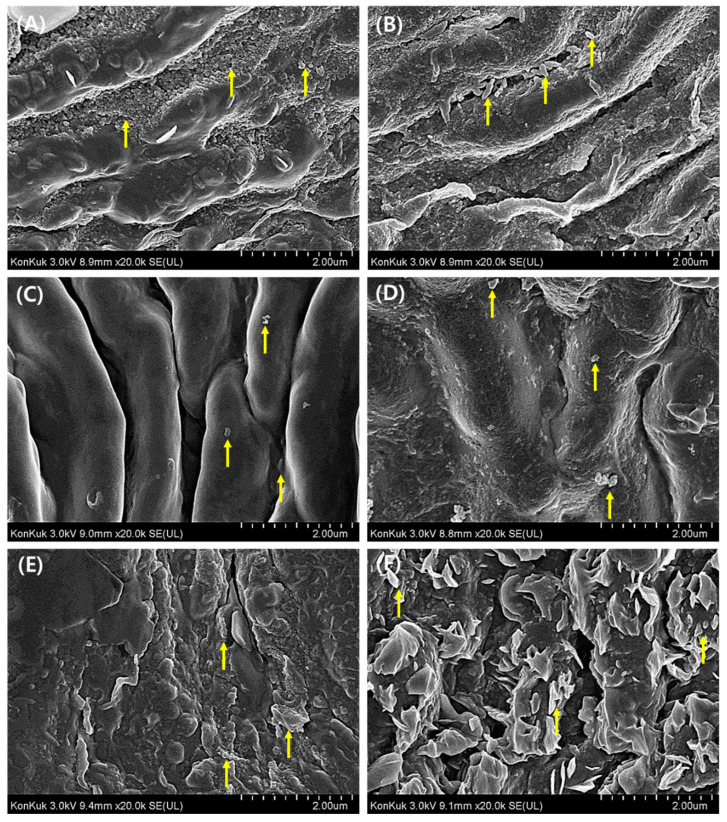
SEM images of adaxial surface of *Ardisia japonica* leaf under CL (continuous light) with (**A**) drought and (**B**) water supply, under LD cycle (light/dark cycle; 14 h/10 h) with (**C**) drought and (**D**) water supply, and under CD (continuous dark) with (**E**) drought and (**F**) water supply. Symbol (↑) indicates the distributed particulate matter.

**Figure 5:**
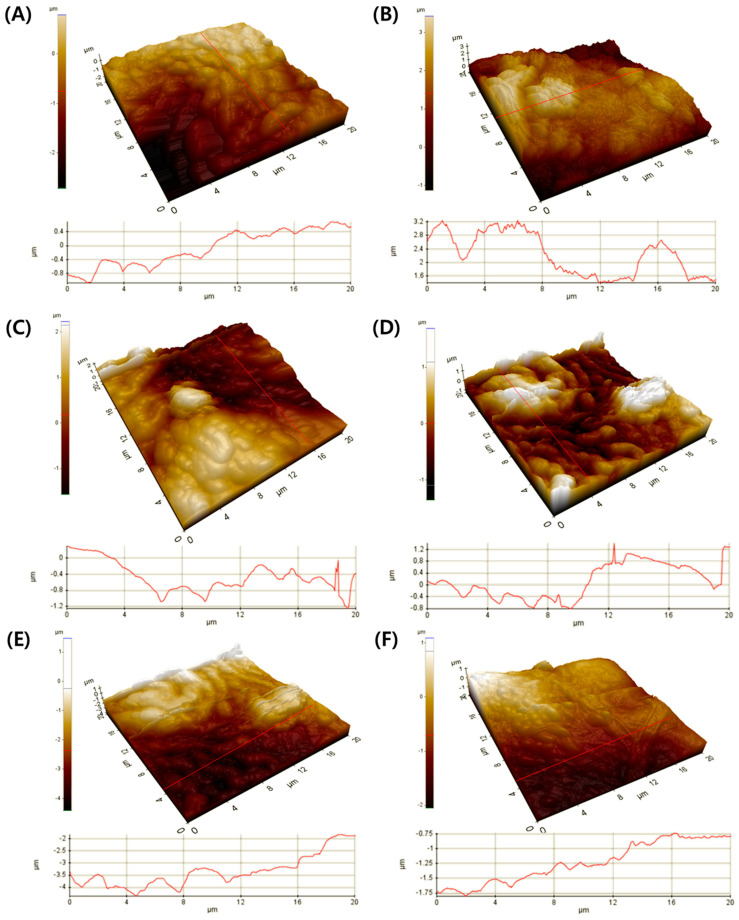
Atomic force microscopy characterization (surface height maps and 3D profiles) of adaxial surface of *Ardisia japonica* leaf under CL (continuous light) with (**A**) drought and (**B**) water supply, under LD cycle (light/dark cycle; 14 h/10 h) with (**C**) drought and (**D**) water supply, and under CD (continuous dark) with (**E**) drought and (**F**) water supply.

The SEM images of abaxial and adaxial surface of *A. japonica* leaf are shown in Figure 3 and Figure 4, respectively. Overall, the leaf stomata were closed under all illumination conditions (CL, LD cycle, and CD) during drought (Figure 3A,C,E). However, the stomata opened in the presence of water (Figure 3B,D,F). Additionally, most PM2.5 was distributed near the opened stomata in the presence of water compared to the drought condition, as shown in Figure 3. These findings may likely indicate PM2.5 uptake during the stomatal opening in the presence of water. Nevertheless, the RE of PM2.5 increased only under the drought condition (Figure 2A,B). These results indicate that several mechanisms, such as uptake by leaf stomata and adsorption on the leaf surface, are likely involved in the removal of PM2.5. As shown in Figure 3A,C, fine and coarse PMs were captured on the leaf surface, and many ripples were observed due to dried leaf. However, fine PM was mostly observed near leaf stomata under water supply condition (Figure 3B,D). These PMs were broadly distributed on the leaf surface under drought conditions as compared to those under water supply. The variations in adaxial surface morphology and increased roughness of *A. japonica* leaf under drought conditions with CL and LD cycle conditions were revealed by SEM and AFM analyses of leaf cross-section (Figure 4 and Figure 5). As shown in Figure 4A,C, the ripples indicating the roughness also appeared on the adaxial surface. In addition, the wax layer was formed on the surface and ripples, and these phenomena may be associated with PM2.5 capture. In particular, the difference in the roughness, as indicated by the leaf curvature, under water and drought conditions was evidently observed from the section of leaf (Figure 5). The leaves were entirely curved under drought conditions with CL, as shown in Figure 5A. These phenomena indicate that the influence of watering conditions on PM2.5 removal can be associated with the changes of the leaf surface, which may result in the capture of PM2.5.

### 3.4. Chemical Evaluation of Leaf Surface for PM2.5 Removal

**Figure 6:**
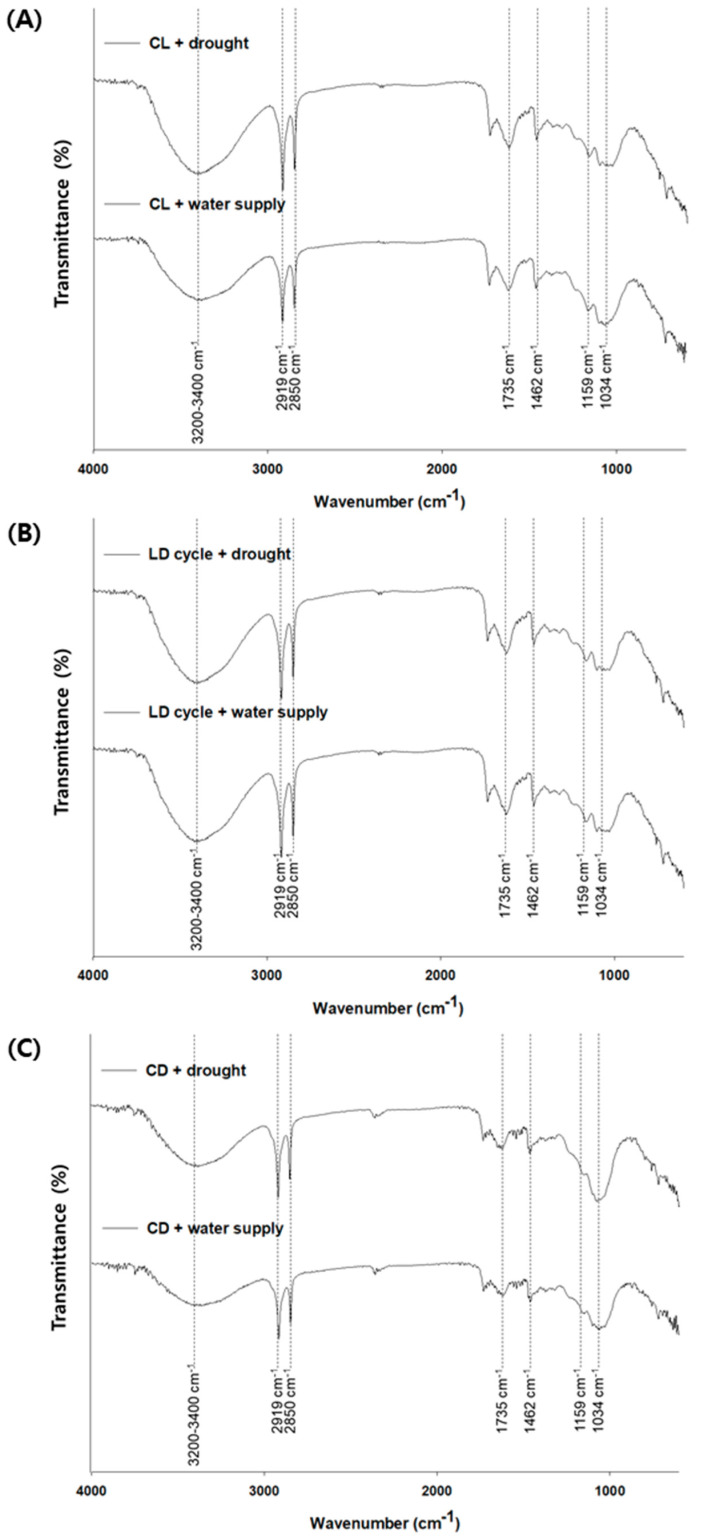
Fourier transform infrared spectra of adaxial surface of *Ardisia japonica* leaf under (**A**) CL (continuous light), (**B**) LD cycle (light/dark cycle; 14 h/10 h), and (**C**) CD (continuous dark) with drought and with water supply.

**Figure 7:**
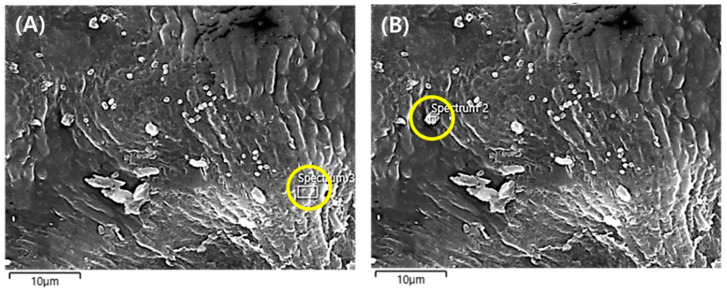
SEM images of (**A**) adaxial surface layer of *Ardisia japonica* leaf and (**B**) particle on the leaf surface under CL (continuous light) with drought.

The accumulated RE of PM2.5 and morphological changes under different watering and illumination conditions are described in Section 3.3. Additionally, the variations in the wax content on the leaf surface were investigated under different watering (water supply and drought) and illumination conditions (CL, LD cycle, and CD) via FTIR analysis (Figure 6). Several peaks were revealed in the area ranging between 600 and 4000 cm^−1^; prominent peaks were observed at 1034, 1159, 1462, 1735, 2850, 2919, and 3200–3400 cm^−1^ that corresponded to C–O (glycosidic bond of polysaccharides), cutin, C–H, cutin, long-chain aliphatic C–H (symmetric CH_2_), long-chain aliphatic C–H (asymmetric CH_2_), and C–OH, respectively (Figure 6) [10,18]. Among them, C–H (1462 cm^−1^), long-chain aliphatic C–H (symmetric CH_2_) (2850 cm^−1^), long-chain aliphatic C–H (asymmetric CH_2_) (2919 cm^−1^), and C–OH (3200–3400 cm^−1^) were associated with the wax layer [10]. The transmittance of all wax-related peaks increased on the leaf surface, and the peak intensity was the highest under drought conditions with CL. However, peak intensities and wax pattern on the leaf surface under LD cycle conditions were almost identical (Figure 6). Regarding the CO_2_ changes mentioned above, the metabolisms might be different compared to other conditions (i.e., CL and CD). In addition, various elemental compositions including Ca, S, and Pt were observed in the captured particle, implying that natural PM2.5 capture was under CL conditions with drought (Figure 7 and Table 1). These results indicate that PM2.5, including various natural particles, can be adsorbed by wax on the curvatures.

### 3.5. Comparison of PM2.5 Removal between H. helix and A. japonica

**Figure 8:**
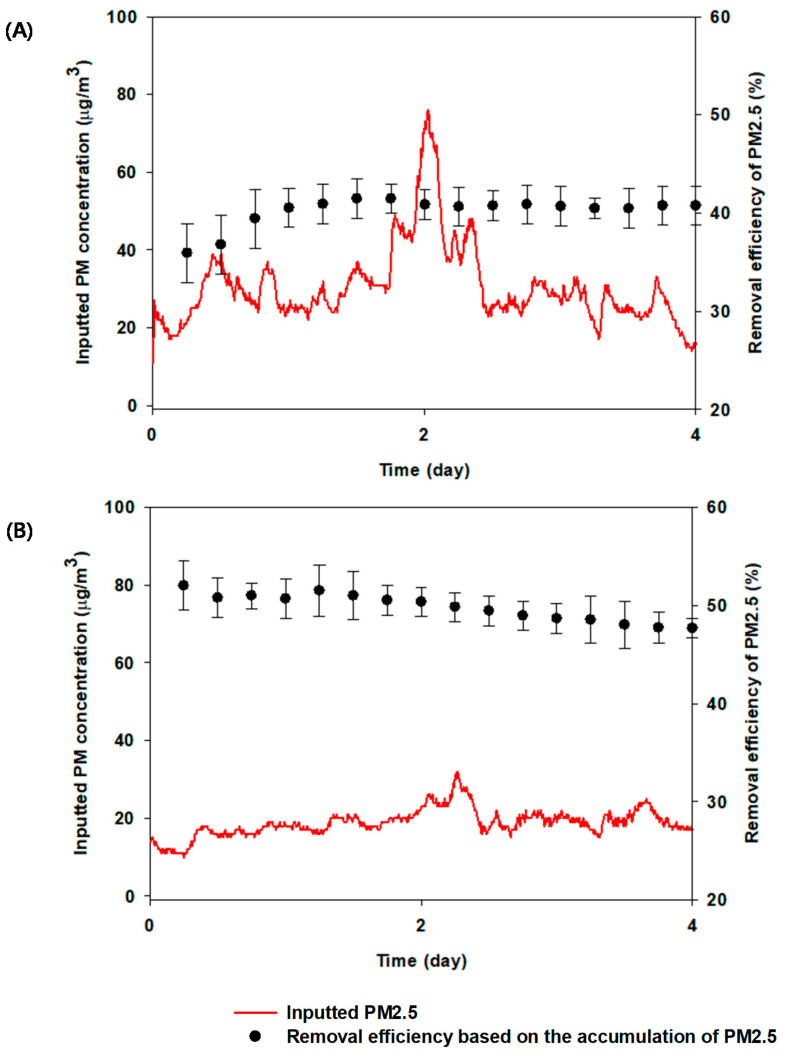
Removal efficiency of PM2.5 by the indoor plant-based biofilter system containing *Hedera helix* as different plant under CL (continuous light) with (**A**) drought and (**B**) water supply. The error bars represent standard deviation.

Higher PM2.5 RE of *A. japonica,* a woody plant, used in this study was demonstrated under drought with CL condition as mentioned above. Its comparison to *H. helix,* as a different woody plant, was conducted to assess the biofilter system under the same watering (i.e., drought and water supply) and CL illumination conditions. According to the results shown in Figure 8A, the RE of PM2.5 increased during 2 days under drought with CL condition, reaching an exponential phase thereafter. However, in the presence of water and CL, the overall RE of PM2.5 decreased (Figure 8B). This may be attributed to the leaf roughness and wax production on the surface of leaf during drought, as observed in *A. japonica* woody plant. In addition, the PM2.5 RE under water supply condition was higher than that under drought. This result may indicate that PM2.5 removal depends on the type of plant, PM concentration, and so on. Based on these results, a temporary drought condition in the soil, as stress, can be a potential and robust strategy in plant-based biofilter systems for enhancing PM2.5 removal.

## 4. Discussion

In this study, the RE of PM2.5 and VOCs increased under drought and constant light (CL) conditions. The results indicate that watering and illumination conditions for the plant based-biofilter system may influence the PM2.5 removal. The stomata of plants are generally closed under drought conditions [13]. Therefore, these phenomena may be associated with other functions of plants for the PM2.5 removal. According to the previous studies, various ecological modifications were evident (e.g., presence of wax and roughness) on the leaf surface under drought conditions [14,19]. Thus, in the present study, higher PM2.5 removal during drought was governed by adsorption rather than uptake. The removal of many inorganic and organic pollutants containing PMs depends on the various factors such as size and type of particle, plant species, environmental conditions (e.g., air temperature, humidity, light), soil, and water [20]. There could be two major mechanisms including adsorption by cuticle and uptake by opened stomata under the optimized conditions in plants cultivation, as reported in the previous literature [21]. Although the portion of PM removal between the adsorption and uptake cannot definitely be elucidated in this study, the increase of wax and roughness by drought can be one of possible approaches. As reported in a previous study, various PMs (21–34 μg/m^3^) such as elemental carbon, organic carbon, sulfate, nitrate, Na, K, Ca, Mg, etc. were observed in Seoul, Republic of Korea, similar to the results in this study (Table 1) [22]. In particular, various elemental compositions including Ca, S, and Pt were captured on the surface of plant leaf. The results may indicate that the elements can be captured selectively based on the properties of plant leaf. According to a previous study, the chemical affinity between the property (i.e., different wax form) of plant leaf and PMs can lead to transport to the stomata [14]. Moreover, illumination may likely contribute to PM2.5 removal. Similar to the results of this study, Kwon et al. (2018) reported that higher light intensity (60 μmol/m^2^/s) influenced the PM removal by *Spathiphyllum* spp., and the removal by *Dieffenbachia amoena* was significantly different between light conditions (30 and 60 μmol/m^2^/s) and dark condition (0 μmol/m^2^/s), resulting in photosynthesis [23].

The accumulated RE of VOCs observed a similar trend as that of PM2.5. Although drought conditions revealed closed stomata, the higher VOCs removal may likely be due to the presence or increase of wax on the leaf surface; previous studies have reported the removal of benzene using wax [15,16].

In contrast, the RE patterns of CO_2_ varied from that of PM2.5 and VOCs. The increase and decrease in CO_2_ is mainly associated with photosynthesis (under illumination) and plant respiration [17]. As reported by Carmo et al. (2012), photosynthesis is considerably sensitive to drought and heat stress due to Rubisco [17]. Moreover, the increase in CO_2_ RE with LD cycle is elucidated; the plants might be adapted to LD cycle, which implies a normal condition for plant growth against a harsh environment [24].

Based on the SEM images of *A. japonica* leaf, the stomata were closed during drought. This phenomenon may be attributed to water preservation under drought conditions [13]. The increase in PM2.5 RE appeared under the drought condition. Therefore, there is a certain mechanism, such as adsorption on the leaf surface, to eliminate PM2.5. The previous studies have reported the changes in morphology and compositions on the leaf surface, such as leaf roughness and wax due to drought, which induced an increase in PM2.5 removal [14,19].

According to the FTIR analysis results, the intensity of all wax-related peaks increased under drought conditions with CL. These findings corroborate the increase in wax on the surface of leaf under the drought condition, as reported in the previous studies [25,26]. Popek et al. (2013) and Räsänen et al. (2014) indicated that the wax layers of coniferous tree species and *Picea abies* (L.) Karst. had a significant role in PM capture [25,27]. Based on the previous studies, the increase in wax could likely increase the PM2.5 removal, as observed in the present study [25,27]. Consequently, the RE of PM2.5 and VOCs increased during drought; these ecological modifications were also associated with PM2.5 removal. In particular, we found that there existed higher roughness and wax layer during drought under CL condition. Based on these results, drought can be temporarily induced in the soil as stress, which can be a potential strategy in enhancing the removal of PM.

## 5. Conclusions

For a plant-based biofilter system, the air circulation rate can be adjusted to cater to the user’s requirement. Nevertheless, certain limitations exist, such as suitable plants species selection, limited space for potted plants, and lower removal efficiency of PM remains. Therefore, this study focuses on the ability of indoor plant-based biofilter system to capture PM2.5 under drought stress with or without illumination. The ecological modifications (i.e., morphological and chemical) on the leaf surface under various conditions were analyzed. The accumulated RE (slope: 2.6569) of PM2.5 was the highest under CL with drought as compared to other illumination conditions (LD cycle’s slope: 1.6040 and CD’s slope: −0.3024) under drought. In particular, the closure of *A. japonica* leaf stomata and the increase in RE of PM2.5 appeared under the drought condition. The difference in leaf curvatures, indicating the roughness between water supply and drought conditions, was evidently observed from the cross-section of the leaf. Additionally, the transmittance of all wax-related peaks increased on the surface of leaf, and the intensity of peaks was highest under drought condition with CL. These results indicate that several mechanisms, such as adsorption by roughness and wax on the surface of the leaf, are involved in the removal of PM2.5. Therefore, the operating parameters of indoor plant based-biofilter system such as watering (or drought) and illumination may be applied as a potential strategy for enhancing PM2.5 removal. In a future work, different shapes and properties of plant leaf including long villi, grooves, and pubescent hairs will be applied to the plant based-biofilter system, and the factors engendering the modifications in characteristics will be analyzed.

## Figures and Tables

**Table 1:** Elemental compositions of the adaxial surface layer of *Ardisia japonica* leaf and leaf surface particle under CL (continuous light) with drought.

	C	O	Si	S	Cl	K	Ca	Pd	Pt	Total (wt%)
Surface layer	75.02	8.86	1.20	-	1.63	4.73	0.84	1.62	6.1	100
Particle	57.97	27.71	0.32	3.26	0.33	0.93	4.31	1.16	4.01	100

C: Carbon, O: Oxygen, Si: Silicon, S: Sulfur, Cl: Chlorine, K: Potassium, Ca: Calcium, Pd: Palladium, Pt: Platinum, wt%: weight%.

## Data Availability

Not applicable.

## Supplementary Materials

The following are available online at https://www.mdpi.com/article/10.3390/plants10122761/s1, Figure S1: Removal efficiency of PM2.5, CO_2_, and VOCs by the plant based-biofilter system containing *Ardisia japonica* as woody plants under (A) CL (continuous light), (B) LD cycle (light/dark cycle; 14 h/10 h), and (C) CD (continuous dark) with water supply during 10 days in parallel. The error bars represent standard deviation (S.D.), Figure S2. Removal efficiency of PM2.5, CO_2_, and VOCs by the plant based-biofilter system containing *Ardisia japonica* as woody plants under (A) CL (continuous light), (B) LD cycle (light/dark cycle; 14 h/10 h), and (C) CD (continuous dark) with drought during 6 days in parallel. The error bars represent standard deviation (S.D.), Figure S3. Removal efficiency of PM2.5, CO_2_, and VOCs by the filter system without plants as control experiment during 6 days. The error bars represent standard deviation (S.D.).
