# Supplementary material for: Morphological and Chemical Evaluations of Leaf Surface on Particulate Matter2.5 (PM2.5) Removal in a Botanical Plant-Based Biofilter System
Source: Plants (Basel). 2021 Dec 14;10(12):2761. doi: 10.3390/plants10122761 (PMC8708160; doi:10.3390/plants10122761)
Supplement: Supplementary file 1 [file plants-10-02761-s001.zip › plants-1497784-supplementary.pdf]

Supplementary data

Morphological and chemical evaluation of leaf surface on particulate matter2.5 (PM2.5) removal in a botanical plant based-biofilter system

Yong-Keun Choi [1], Hak Jin Song [1], Jeong Wook Jo [1], Seong Won Bang [2], Byung Hoon Park [2], Ho Hyun Kim [3], Kwang Jin Kim [4], Na Ra Jeong [4], Jeong Hee Kim [4] and Hyung Joo Kim [1,*]

[1] Department of Biological Engineering, Konkuk University, Seoul 05029, Republic of Korea; dragonrt@konkuk.ac.kr (Y-K.C.); hjeda11@naver.com (H.J.S.); jjw9802@naver.com (J.W.J); hyungkim@konkuk.ac.kr (H.J.K)

[2] Garden4u Co., Gyeonggi-do 15524, Republic of Korea; garden4u_comp@naver.com (S.W.B); byonghpark@naver.com (B.H.P)

[3] Department of Integrated Environmental System, Pyeongtaek University, Pyeongtaek 17869, Republic of Korea; ho4sh@ptu.ac.kr (H.H.K)

[4] Urban Agriculture Research Division, National Institute of Horticultural and Herbal Science, Chun Joo 54875, Republic of Korea; kwangjin@korea.kr (K.J.K); jnr202@korea.kr (N..RJ); kimjhee@korea.kr (J.H.K)

* Correspondence: hyungkim@konkuk.ac.kr; Tel.: +82-02-2049-6111

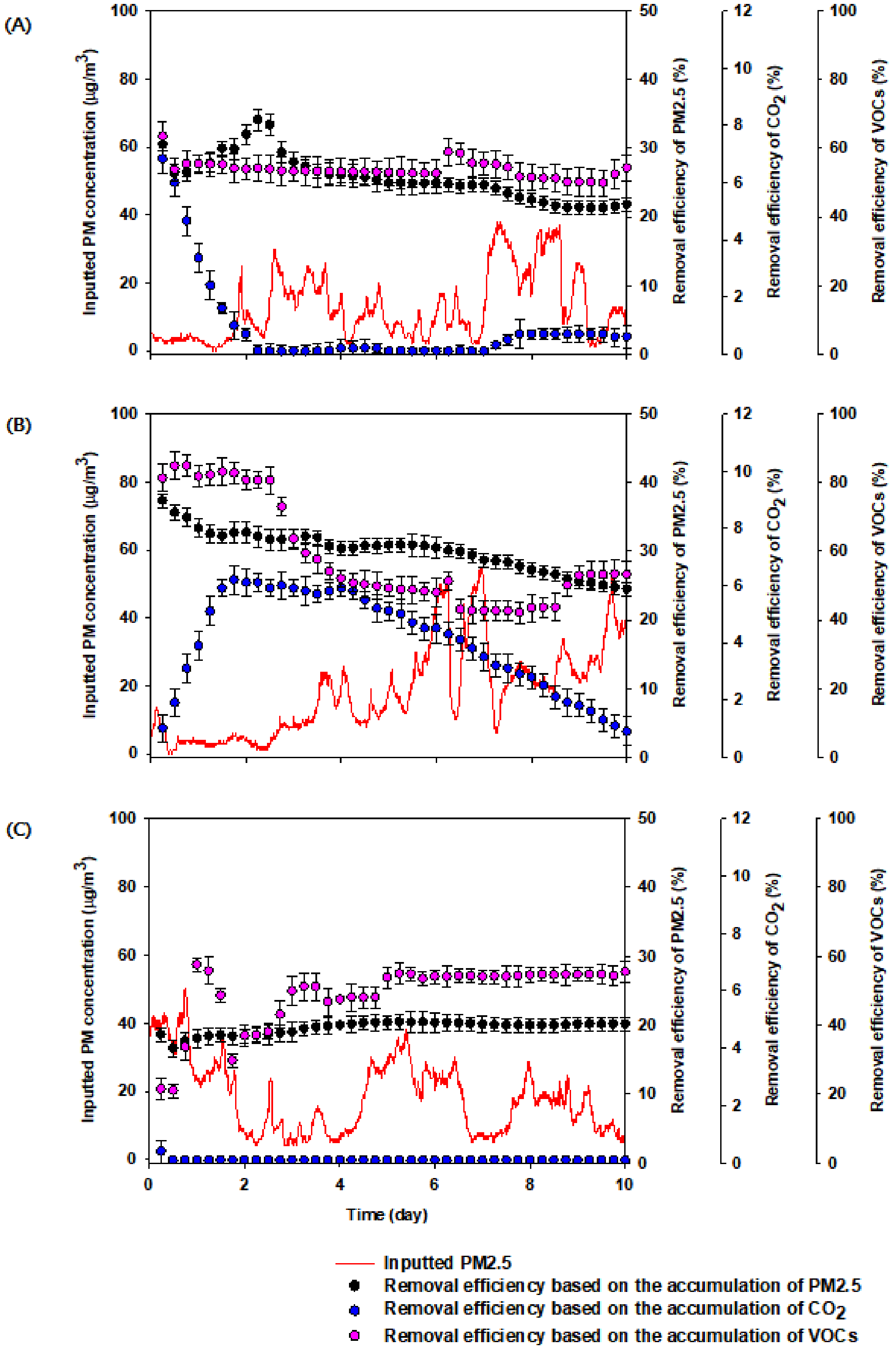

Figure S1. Removal efficiency of PM2.5, CO_2, and VOCs by the plant based-biofilter system containing *Ardisia japonica* as woody plants under (A) CL (continuous light), (B) LD cycle (light/dark cycle; 14 h/10 h), and (C) CD (continuous dark) with water supply during 10 days in parallel. The error bars represent standard deviation (S.D.).

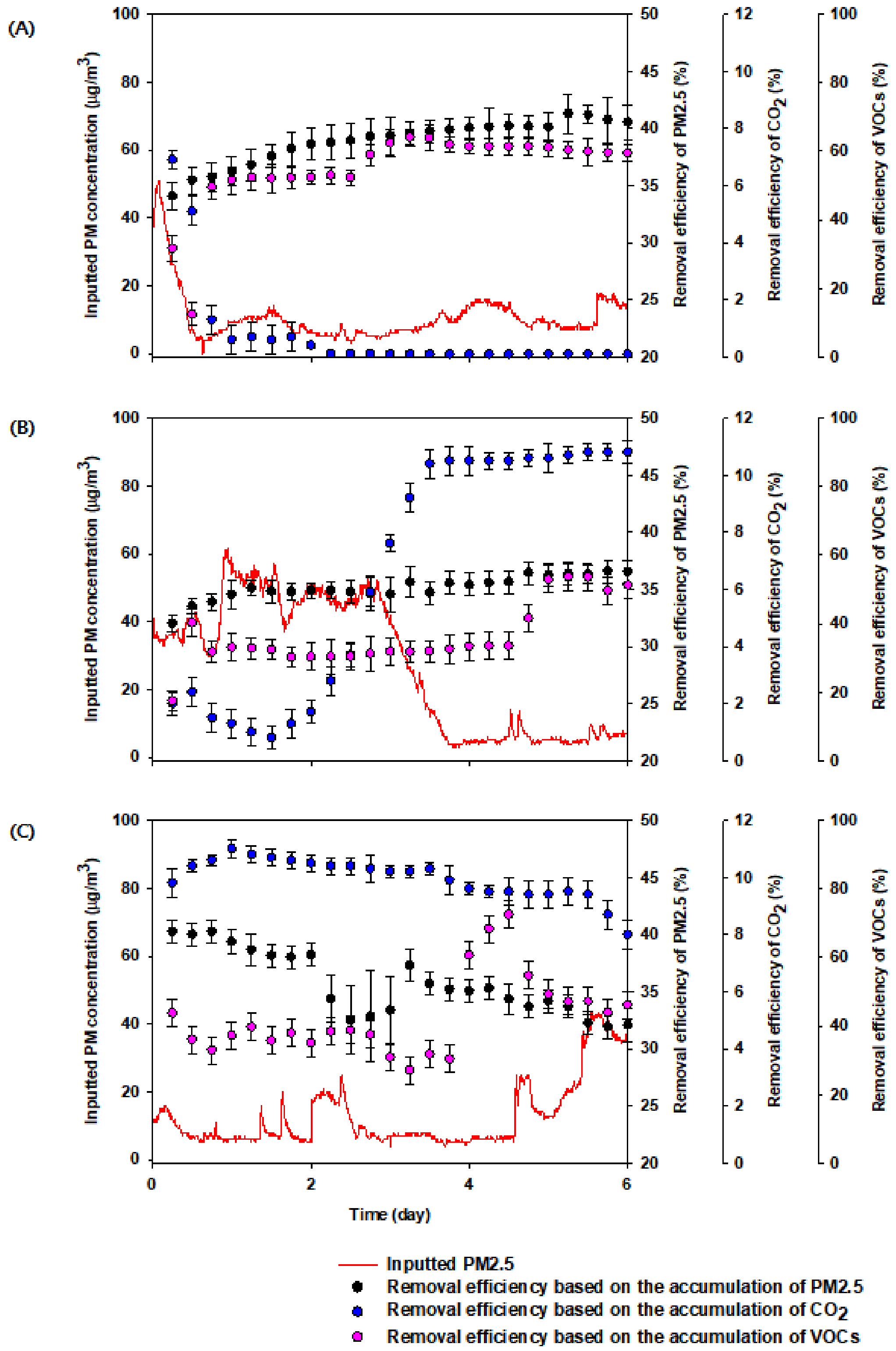

Figure S2. Removal efficiency of PM2.5, CO_2, and VOCs by the plant based-biofilter system containing *Ardisia japonica* as woody plants under (A) CL (continuous light), (B) LD cycle (light/dark cycle; 14 h/10 h), and (C) CD (continuous dark) with drought during 6 days in parallel. The error bars represent standard deviation (S.D.).

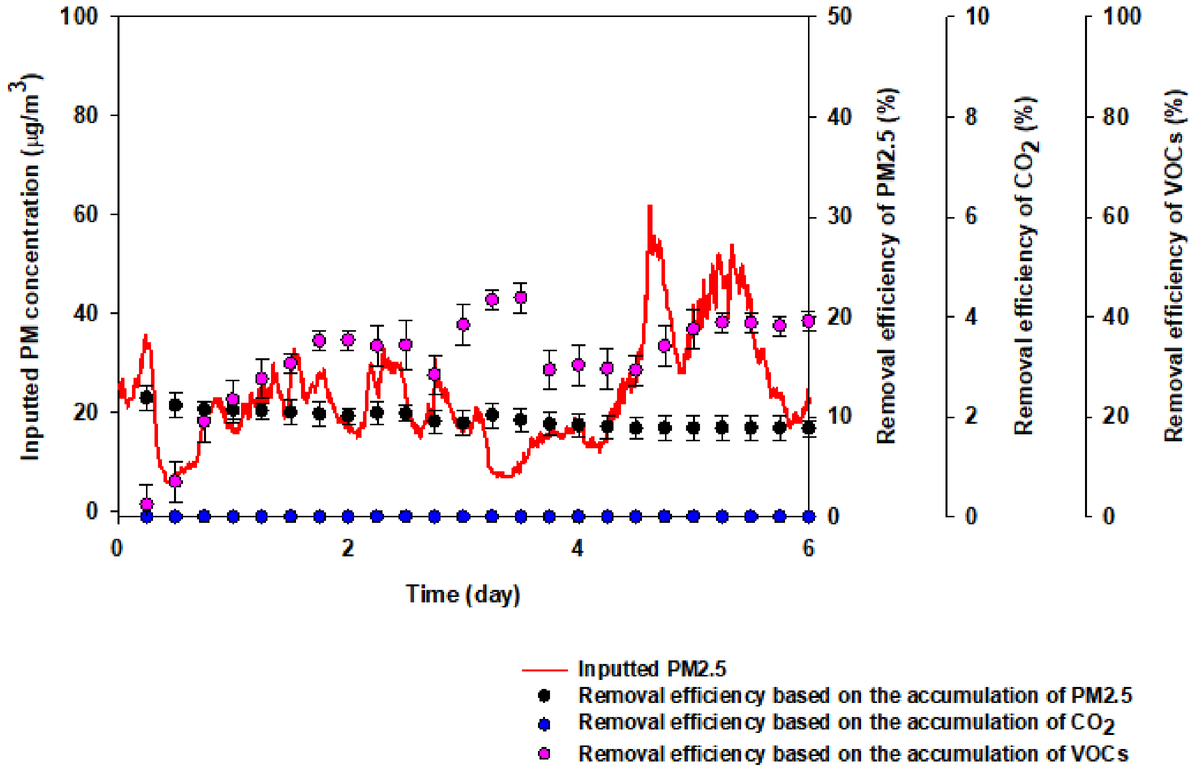

Figure S3. Removal efficiency of PM2.5, CO_2, and VOCs by the filter system without plants as control experiment during 6 days. The error bars represent standard deviation (S.D.).
